# Partner testing with HIV self‐test distribution by Ugandan pregnant women living with HIV: a randomized trial

**DOI:** 10.1002/jia2.26156

**Published:** 2023-09-07

**Authors:** Andrew Mujugira, Agnes Nakyanzi, Deborah Donnell, Jade Boyer, Gabrielle Stein, Michelle Bulterys, Faith Naddunga, Juliet Kyomugisha, Juliet E. Birungi, Paul Ssendiwala, Rogers Nsubuga, Timothy R. Muwonge, Joshua Musinguzi, Monisha Sharma, Connie L. Celum

**Affiliations:** ^1^ Infectious Diseases Institute Makerere University Kampala Uganda; ^2^ Fred Hutch Cancer Center Seattle USA; ^3^ Department of Global Health University of Washington Seattle Washington USA; ^4^ Department of Epidemiology University of Washington Seattle Washington USA; ^5^ AIDS Control Program Ministry of Health Kampala Uganda; ^6^ Departments of Global Health, Medicine, and Epidemiology University of Washington Seattle Washington USA

**Keywords:** HIVST, secondary distribution, PrEP, ART, male partners, Africa

## Abstract

**Introduction:**

Secondary distribution of HIV self‐tests (HIVST) by HIV‐negative pregnant women to male partners increases men's testing rates. We examined whether this strategy promotes male partner testing for pregnant women living with HIV (PWLHIV).

**Methods:**

We conducted an open‐label individually randomized trial in Kampala, Uganda, in which PWLHIV ≥18 years who reported a partner of unknown HIV status were randomized 2:1 to secondary distribution of HIVST for male partner(s) or standard‐of‐care (SOC; invitation letter to male partner for fast‐track testing). Women were followed until 12 months post‐partum. Male partners were offered confirmatory HIV testing and facilitated linkage to antiretroviral treatment (ART) or oral pre‐exposure prophylaxis (PrEP). Using intention‐to‐treat analysis, primary outcomes were male partner testing at the clinic and initiation on PrEP or ART evaluated through 12 months post‐partum (ClinicalTrials.gov, NCT03484533).

**Results:**

From November 2018 to March 2020, 500 PWLHIV were enrolled with a median age of 27 years (interquartile range [IQR] 23–30); 332 were randomized to HIVST and 168 to SOC with 437 PWLHIV (87.4%) completing 12 months follow‐up post‐partum. Of 236 male partners who tested at the clinic and enrolled (47.2%), their median age was 31 years (IQR 27–36), 45 (88.3%) men with HIV started ART and 113 (61.1%) HIV‐negative men started PrEP. There was no intervention effect on male partner testing (hazard ratio [HR] 1.04; 95% confidence interval [CI]: 0.79–1.37) or time to ART or PrEP initiation (HR 0.96; 95% CI: 0.69–1.33). Two male partners and two infants acquired HIV for an incidence of 0.99 per 100 person‐years (95% CI: 0.12–3.58) and 1.46 per 100 person‐years (95% CI: 0.18%–5.28%), respectively. Social harms related to study participation were experienced by six women (HIVST = 5, SOC = 1).

**Conclusions:**

Almost half of the partners of Ugandan PWLHIV tested for HIV with similar HIV testing rates and linkage to ART or PrEP among the secondary distribution of HIVST and SOC arms. Although half of men became aware of their HIV serostatus and linked to services, additional strategies to reach male partners of women in antenatal care are needed to increase HIV testing and linkage to services among men.

## 1 INTRODUCTION

Pregnant women living with HIV (PWLHIV) are more likely to achieve viral suppression (VS) and complete the prevention of mother‐to‐child HIV transmission (PMTCT) cascade when their partners are tested and engaged in care [1]. PWLHIV who had not disclosed their HIV status to male partners in Malawi were more likely to have suboptimal antiretroviral treatment (ART) adherence and transmit HIV to infants than women who had disclosed [2]. Women's non‐disclosure is challenging due to their fear of intimate partner violence (IPV), stigmatization and loss of relationship, housing, and economic support impacting post‐partum ART continuation and sustained VS [3].

Men in East and Southern Africa have significantly lower HIV testing coverage, link to HIV care at more advanced disease stages, and are less likely to be on ART and be virally suppressed than women [4]. HIV testing rates among partners of PLWHIV in sub‐Saharan Africa (SSA) range from 6.1% in Chad to 98.1% in Rwanda where PMTCT programmes offer “opt‐out” testing with strong encouragement for male partners to test [5]. Reasons for men's reluctance to test include fear of knowing their HIV status, and men's belief that their HIV status is the same as their partners [6]. Male partners of PWLHIV are a high‐priority population who benefit from ART if they test positive or HIV pre‐exposure prophylaxis (PrEP) if negative and in serodifferent partnerships [7].

Secondary distribution of HIV self‐testing (HIVST) kits may address men's concerns about attending antenatal care (ANC) clinics for HIV testing [8]. In a pilot of secondary distribution of HIVST among HIV‐negative women in Kenya, significantly higher rates of male partner testing were achieved in the HIVST arm (91%) versus control (52%); couples testing increased by 42% and disclosure by 39% [9]. A randomized trial of women‐delivered HIVST among PWLHIV attending ANC in Malawi found that male partner testing was 1.71 times (95% confidence interval [CI] 1·48–1·98) higher than the standard invitation letter (73% vs. 35% tested) [10]. However, in these studies, HIVST kits were distributed by HIV‐negative women who likely do not face the same barriers and fears as PWLHIV. To evaluate the benefits and risks of secondary distribution of HIVST from PWLHIV, we conducted a randomized trial to evaluate whether secondary distribution of HIVSTs increased male partner HIV testing and initiation of ART or PrEP, without social harms to pregnant women.

## 2 METHODS

### 2.1 Study design

The Obumu Study (“Stronger Together” in Luganda) was an open‐label, individually randomized controlled trial (RCT) of PWLHIV with a partner of unknown HIV status who were recruited from the PMTCT programme at Kitebi Health Center III, a public antenatal clinic in Kampala, Uganda. The intervention was provision of, and training PWLHIV in the use of, HIVST kits through secondary distribution to male partner(s) compared to standard‐of‐care (SOC) invitation letter for fast‐track testing.

This trial is reported in accordance with the Consolidated Standards of Reporting Trials statement [11]. The study was approved by the National HIV/AIDS Research Committee (ARC200), Uganda National Council for Science and Technology (SS4501), and the University of Washington (STUDY00002257).

### 2.2 Participants

**Figure 1:**
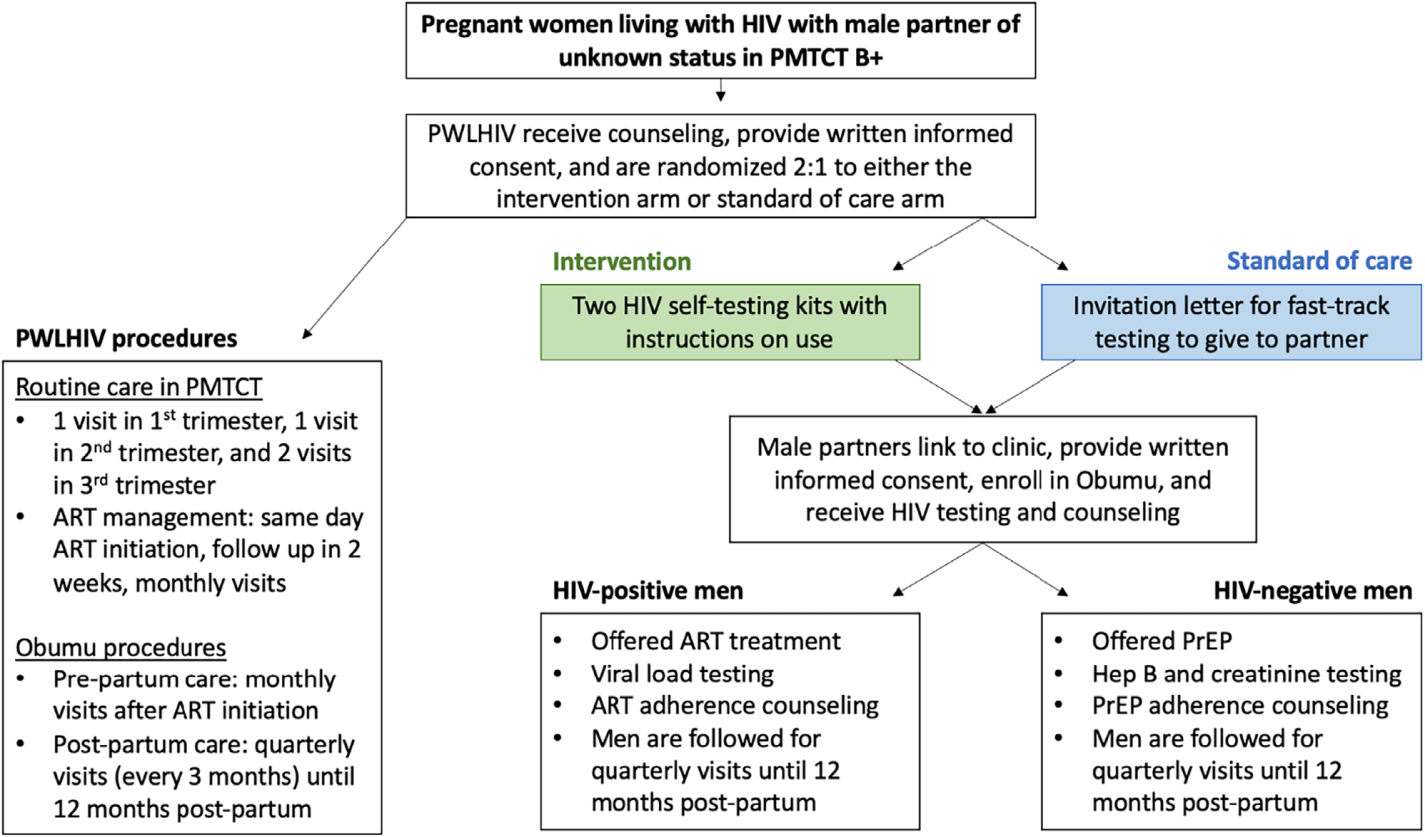
Study procedures for PWLHIV and male partners enrolled in Obumu.

PWLHIV attending ANC at Kitebi clinic were informed about the benefits of male partner testing, post‐partum ART persistence and study participation. Women's baseline IPV risk was assessed using the World Health Organization (WHO) standardized screening tool for clinical diagnosis of IPV [12]. Women were eligible if they were ≥18 years, currently pregnant, reported no IPV and had a partner of unknown status. Eligible women were randomized 2:1 to the intervention (HIVST kits for male partners, referral/transport reimbursement voucher and PrEP/ART educational materials), or SOC (invitation letters to deliver to male partners for fast‐track HIV testing at Kitebi clinic, and PrEP/ART educational materials) (Figure 1). All participants provided written informed consent in English or Luganda and were reimbursed 30,000 Uganda shillings ($8) per study visit.

### 2.3 Randomization and masking

Randomization was open‐label, with variable size blocks, without stratification. The study statistician generated the random allocation sequence using Statistical Analysis Software (SAS) Programming, by blocks in a 2:1 ratio for the intervention (secondary distribution of HIVST) and SOC. Sequentially numbered envelopes corresponding to the random allocation sequence were used to implement randomization immediately upon written consent. Participants and providers were unblinded to arm assignments, but investigators were blinded until the study end.

### 2.4 Procedures

Study staff provided counselling about HIV status disclosure, importance of male partner testing and support for mutual disclosure. Women in the intervention arm were provided a brief HIVST demonstration and given two oral fluid‐based HIVSTs (OraQuick rapid HIV‐1/2) to share with their partners. Written and pictorial instructions in English/Luganda indicated how to perform the test and interpret the results. Women were counselled to assess the risk of IPV when deciding whether and how to introduce HIVST or an invitation letter to partners. PLWHIV were not obligated to distribute self‐tests to partners, particularly those they believed could become violent after introducing HIV testing. Study counsellors discussed with women key messages to use after partner testing, and encouragement for their partner to link to ART/PrEP. Women in the HIVST arm could call a toll‐free 24‐hour helpline if they had questions, needed help explaining counselling messages to their partner or experienced social harms.

Women in the SOC arm were provided a letter to deliver to their partner, inviting him to come to the clinic for fast‐track HIV testing, alone or as a couple. Invitation letters included positively framed messages to men about ART/PrEP benefits, depending on their status, and information on contacting the 24‐hour helpline about results and referrals. Letters had unique numbers to allow linkage of the man's testing to his partner. With the woman's approval, a male counsellor called the male partner if she reported giving him the kit or letter and he had not enrolled after 1 month.

The study aimed to link the male partner to the research clinic, confirm his HIV status and offer ART (if he tested positive) or PrEP (if he tested negative). Lamivudine/tenofovir disoproxil fumarate as PrEP is SOC in Uganda but has not been consistently or widely offered to male partners of PWLHIV. Men were consented to participate for up to 12 months post‐partum (Figure 1). Study staff recommended that HIV‐negative men use PrEP until their partner achieved VS or if they reported additional partners according to national guidelines; women's HIV status was not disclosed to partners without their permission. For men who started PrEP, dried blood spot (DBS) samples were collected at the 3‐ and 6‐month visits for quantification of tenofovir‐diphosphate (TFV‐DP) levels.

Study visit frequency aligned with PMTCT schedules and occurred every 3 months post‐partum through 12 months. Women were asked about their medical history, prior pregnancies, partner's history of HIV testing/HIV status and her assessment of IPV risk from offering him HIVST. Women were counselled about strategies to minimize social harms from HIVST and/or disclosure and referred to social support services if social harms occurred.

Male follow‐up began at their enrolment and ended when their partner reached 12 months post‐partum. Clinic‐based HIV testing for all men was performed using serial rapid tests [13]. HIV‐1 RNA viral load (VL) monitoring was performed at the Uganda Central Public Health Laboratory for PWLHIV at enrolment, 6 and 12 months post‐partum and for men who initiated ART at 6 months and annually thereafter [14]. For men taking PrEP, TFV‐DP levels from the 3‐month visit were quantified from DBS using validated liquid chromatography‐tandem mass spectrometry methods [15].

### 2.5 Outcomes

The primary outcomes were (1) male partners testing and enrolling at the study clinic and (2) male partners initiated on PrEP or ART, evaluated through 12 months post‐partum. The secondary outcome was maternal VS at 12 months post‐partum. Serious adverse events related to PrEP were clinically monitored and reported in accordance with local guidelines.

### 2.6 Statistical analysis

The sample size of 500 provided 90% power to detect an increase in HIV testing from 30% in the SOC arm to 55% in the intervention arm, with a 1:2 randomization ratio and two‐sided alpha = 0.05. The intention‐to‐treat analysis included all women enrolled and randomized. Kaplan−Meier methods and log‐rank tests were used to compare time from the women's enrolment until her male partner was tested in the clinic, by randomization arm (as women had different study duration, depending on the trimester of enrolment). Pointwise confidence intervals for the proportion tested 12 months after the women's enrolment were computed assuming the normal approximation, using the standard error of the estimate from the Kaplan−Meier curve. Cox proportional hazards regression was used to estimate hazard ratios (HRs) and 95% CI. Since the intervention could affect male partner enrolment differentially by HIV status, we assessed the probability of PrEP initiation for all HIV‐negative men, both unconditional and conditional on enrolment. We assessed the difference in PrEP initiation between arms among the (unobserved) true number of HIV‐negative male partners of enrolled pregnant women using a likelihood ratio test. The likelihood of difference in PrEP initiation between arms was computed assuming the probability of an HIV‐negative partner was the same in both arms. Factors associated with maternal VS were assessed using Poisson regression with robust standard errors. Statistical analyses were performed using SAS version 9.4 (SAS Institute). Study data were reviewed every 6 months by an independent data monitoring committee.

## 3 RESULTS

**Figure 2:**
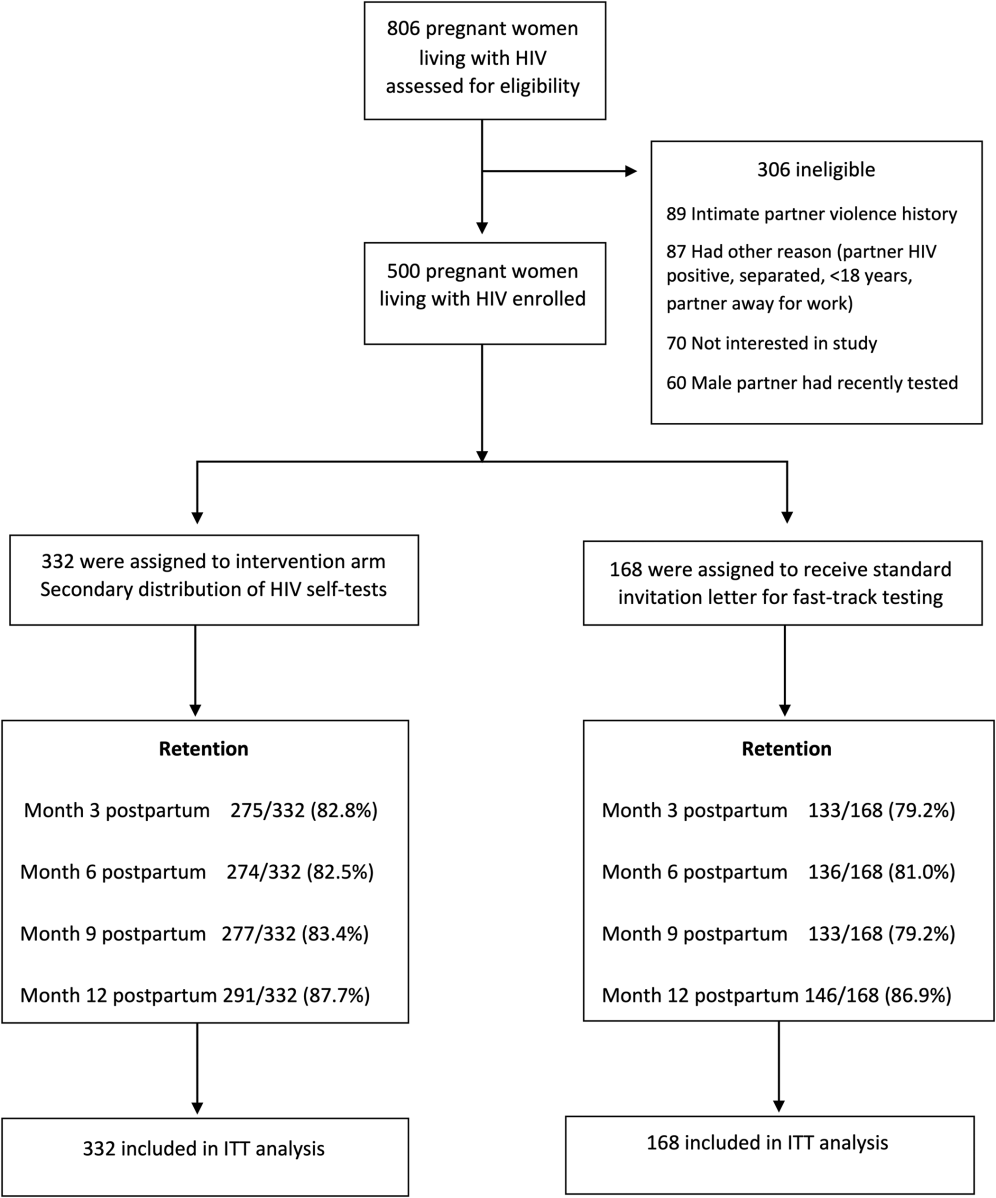
CONSORT diagram for Obumu Study.

Between 14 November 2018, and 19 March 2020, we screened 806 and enrolled 500 PWLHIV; reasons for ineligibility are shown in Figure 2. Of these, 332 (66.4%) and 168 (33.6%) were randomized to HIVST intervention and SOC arms, respectively (Table 1). Across both arms, the median age of women was 27 years (interquartile range [IQR] 23–30), 95% were married and ∼30% were in a polygamous relationship. At enrolment, 96% of women were taking ART and 50% had VS (<50 copies/ml); among those with detectable VL, median VL was <1000 copies/ml (755 and 339 copies/ml in the SOC and HIVST arms, respectively). Among the 498 women followed, the median duration of follow‐up was 38.4 weeks (IQR 34.6–43.2) with a total of 621.4 person‐years of follow‐up. Retention at 12 months post‐partum was 87.4% (437/500): 87.7% (291/332) in the HIVST arm and 86.9% (146/168) in the SOC arm.

**Table 1:** Baseline characteristics of pregnant women living with HIV.

Characteristic	Total, *N* (%)	HIVST, *N* (%)	Letter, *N* (%)
Enrolled	500	332	168
Age; median (IQR)	27 (23–30)	26 (23–30)	27 (23–31)
Marital status			
Currently married	477 (95.4%)	312 (94.0%)	165 (98.2%)
Divorced/separated	5 (1.0%)	5 (1.5%)	0 (0%)
Never married	16 (3.2%)	15 (4.5%)	1 (0.6%)
Polygamous relationship	147 (29.4%)	96 (28.9%)	51 (30.4%)
Number of prior live births			
0	89 (17.8%)	62 (18.7%)	27 (16.1%)
1–2	276 (55.2%)	175 (52.7%)	101 (60.1%)
≥ 3	135 (27.0%)	95 (28.6%)	40 (23.8%)
Trimester at Obumu enrolment			
First trimester	58 (11.6%)	42 (12.7%)	16 (9.5%)
Second trimester	244 (48.8%)	156 (47.0%)	88 (52.4%)
Third trimester	198 (39.6%)	92 (39.3%)	106 (39.8%)
ART use at enrolment			
Current	478 (95.6%)	318 (95.8%)	160 (95.2%)
Started day of enrolment	18 (3.6%)	11 (3.3%)	7 (4.2%)
Not started ART at enrolment	4 (0.8%)	0 (0%)	1 (0.6%)
Plasma viral load at enrolment			
Undetectable	251 (50.2%)	166 (50.0%)	85 (50.6%)
Detectable	242 (48.4%)	163 (49.1%)	79 (47.0%)
Missing	7 (1.4%)	3 (0.9%)	4 (2.4%)
Plasma viral load (copies/ml among women with detectable VL); median (IQR)	389.5 (94.0–5100.0)	339.0 (94.0–3610.0)	755.0 (94.0–5528.0)
Partner ever tested for HIV *reported by women*			
Yes	133 (26.6%)	87 (26.2%)	46 (27.4%)
No	189 (37.8%)	122 (36.7%)	67 (39.9%)
Don't know	178 (35.6%)	123 (37.0%)	55 (32.7%)
Male partner's HIV status from prior test^a^			
HIV positive	2 (1.5%)	2 (2.3%)	0 (0%)
HIV negative	126 (94.7%)	81 (93.1%)	45 (97.8%)
Don't know	5 (3.8%)	4 (4.6%)	1 (2.2%)

^a^ Male partner's HIV status is reported among women who reported knowing if her partner had ever tested for HIV.

**Figure 3:**
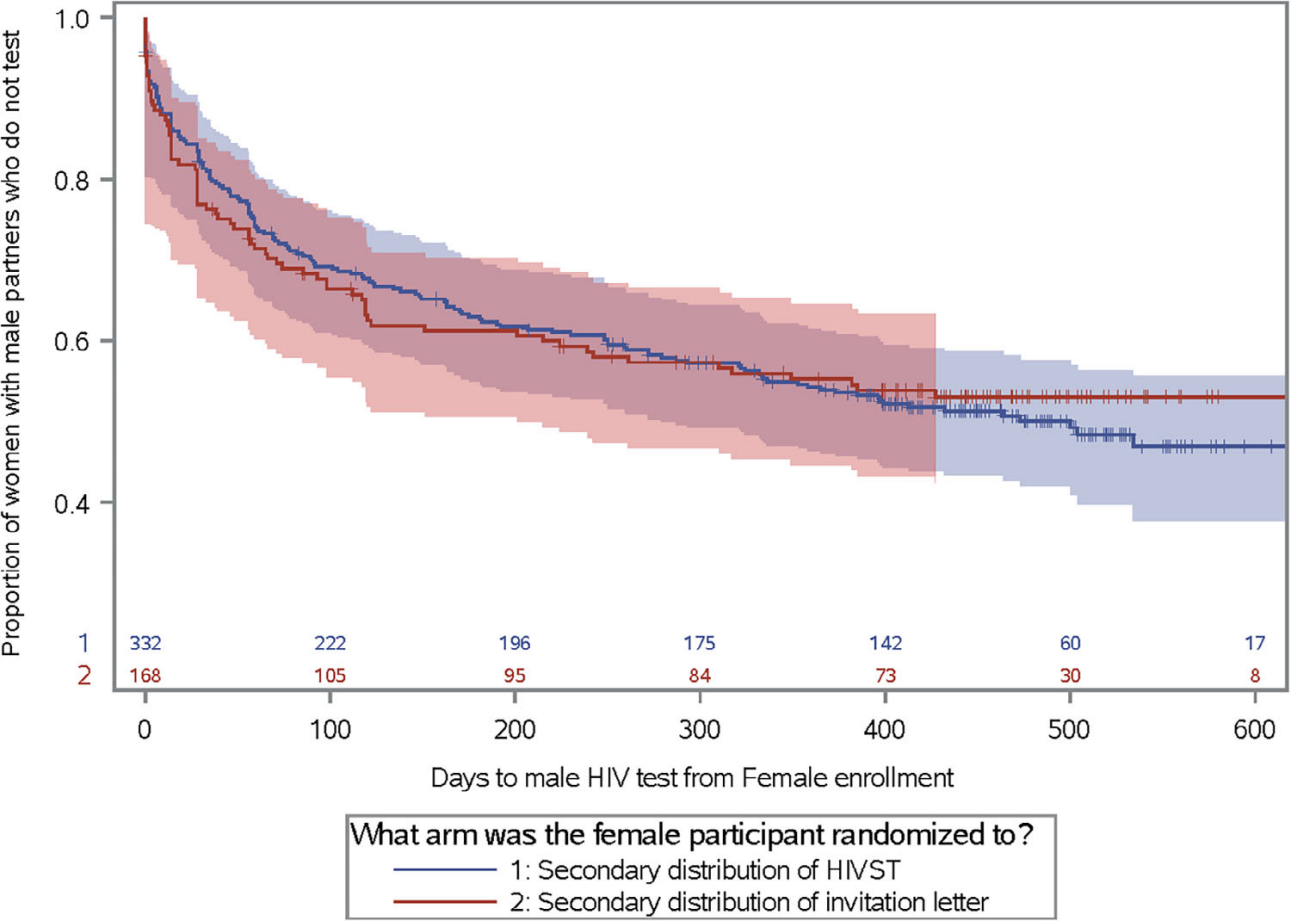
Time to male partner enrolment from randomization of female partner.

The median age of men was 31 years (IQR 27–36), 52% were ≥5 years older than their female partner and the median duration of partnership was 38.0 months (IQR 21.0–73.0) (Table 2). Nearly 60% reported previously testing for HIV, with a median of 5.8 months since their last test (IQR 3.1–11.7). Among men who previously tested for HIV, nearly thrice as many in the HIVST arm previously tested HIV positive compared to the SOC arm (11.7% vs. 4.3%). At enrolment, 21.6% (51/236) of men tested HIV positive; 23.6% and 17.3% in the HIVST and SOC arms, respectively. Overall, 236 male partners tested for HIV: 48.5% (161/332) in the intervention and 44.6% (75/168) in the SOC arm. There was no difference in the proportion of men testing for HIV by randomization arm (HR 1.04; 95% CI: 0.79–1.37) (Figure 3). By 12 months after the female partner's enrolment, 33.2% of male partners in the intervention and 33.3% in the SOC arm had tested for HIV at the clinic (Difference = 0.1%; 95% CI: −9.2 to 9.4). A secondary outcome of women's self‐reported delivery of HIVST or invitation letter for fast‐track testing was similar by arm: HIVST (81.6%) and invitation letters (81.5%) (Table 3).

**Table 2:** Women's self‐reported delivery of HIVST or invitation letter

	HIVST, *N* (%)		Letter, *N* (%)	
	Male partners enrolled	Male partners did not enroll	Male partners enrolled	Male partners did not enroll
Enrolled women	161	171	75	93
Female reported giving partner HIVST/letter	143/161 (88.8%)	128/171 (74.9%)	65/75 (86.7%)	72/93 (77.4%)
Female reported partner tested at clinic or HIVST	109/143 (76.2%)	75/128 (58.6%)	20/65 (30.8%)	8/72 (11.1%)
Female self‐report about male partner's HIV result				
HIV positive	17/109 (15.6%)	22/75 (29.3%)	1/20 (5.0%)	3/8 (37.5%)
HIV negative	84/109 (77.1%)	48/75 (64.0%)	16/20 (80.0%)	4/8 (50.0%)
Don't know	8/109 (7.3%)	5/75 (6.7%)	3/20 (15.0%)	1/8 (12.5%)
Male HIV status at enrolment Among men enrolled in Obumu Study				
HIV positive	38/161 (23.6%)	−	13/75 (17.3%)	−
HIV negative	123/161 (76.4%)	−	62/75 (82.7%)	−

**Table 3:** Demographic and baseline characteristics of male partners who tested and enrolled, overall and by randomization arm of female partner

	Total, *N* (%)	HIVST, *N* (%)	Letter, *N* (%)
Enrolled	236	161	75
Age			
18–24	24 (10.2%)	16 (9.9%)	8 (10.7%)
25–29	67 (28.4%)	42 (26.1%)	25 (33.3%)
30–34	65 (27.5%)	46 (28.6%)	19 (25.3%)
≥35	80 (33.9%)	57 (35.4%)	23 (30.7%)
Age; median (IQR)	31 (27–36)	32 (28–36)	31 (27–36)
Age difference between partners			
Male greater than 5 years older	123 (52.1%)	90 (55.9%)	33 (44.0%)
Male 0–5 years older	81 (34.3%)	50 (31.1%)	31 (41.3%)
Male 0–5 years younger	20 (8.5%)	16 (9.9%)	4 (5.3%)
Male at least 5 years younger	12 (5.1%)	5 (3.1%)	7 (9.3%)
Partnership duration (months), median (IQR)	38.0 (21.0, 73.0)	36.0 (24.0, 77.0)	38.0 (18.0, 72.0)
Men reported ever testing for HIV			
Yes	140 (59.3%)	94 (58.4%)	46 (61.3%)
No	74 (31.4%)	51 (31.7%)	23 (30.7%)
Not sure	21 (8.9%)	15 (9.3%)	6 (8.0%)
No answer	1 (0.4%)	1 (0.6%)	0 (0%)
Months since last HIV test, median (IQR)	5.8 (3.1, 11.7)	5.4 (2.6, 11.5)	5.9 (3.5, 17.4)
Results of most recent HIV test among men who previously tested			
HIV positive	13 (9.3%)	11 (11.7%)	2 (4.3%)
HIV negative	126 (90.0%)	83 (88.3%)	43 (93.5%)
No answer	1 (0.7%)	0 (0%)	1 (2.2%)
Reported additional partners	105 (44.5%)	76 (47.2%)	29 (38.7%)

**Figure 4:**
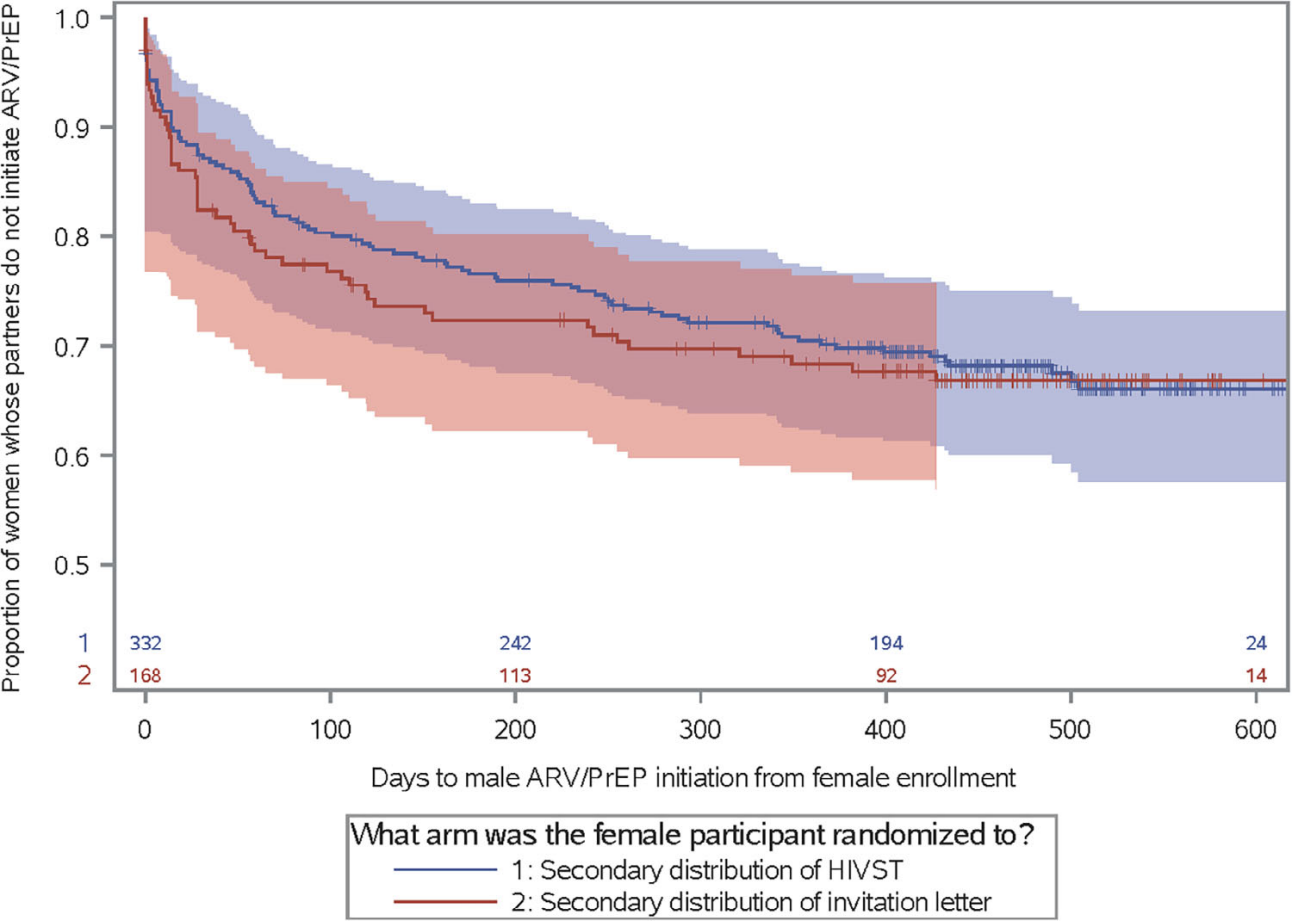
Proportion of men who initiated ARV or PrEP by study arm.

Of the 51 newly identified male partners living with HIV, 86.9% and 92.3% of men in the intervention and SOC arms started ART before or during the study. Among 185 male partners without HIV, 58.6% and 66.1%, respectively, of male partners of women randomized to the intervention and SOC arms started PrEP. There was no difference in male partner ART/PrEP initiation by randomization arm: 32.2% (105/326) and 34.0% (53/156), respectively (HR 0.96; 95% CI: 0.69–1.33) (Figure 4). There were two seroconversions in men in the HIVST arm (HIV incidence, 0.99; 95% CI: 0.12–3.58 per 100 person‐years). For both men, their female partners had HIV RNA levels <50 copies/ml at baseline and study exit. One man initiated PrEP and tested HIV positive at the 12‐month visit; his TFV‐DP levels were high (831.62 fmol/punch) at the 3‐month visit.

At baseline, 159 women (31.8%) reported HIV status disclosure to their partner—34.8% and 26.8% of women in the HIVST and SOC arms, respectively (Table 4). By delivery, an additional 13% of women had disclosed, and by 12 months post‐partum, 294 women (59%) had disclosed their status, with no differences by arm. Of the 428 women (85.6%) with VL available at 12 months post‐partum, 80.8% (346/428) had suppressed HIV RNA levels, with no difference by arm (84.2% vs. 76.4%, relative risk [RR] 1.10; 95% CI: 0.88–1.38).

**Table 4:** Woman's disclosure of HIV status to her male partner

Visit	Total, *N* (%)	Secondary distribution of HIVST arm *N* (%)	Invitation letter for fast‐track testing arm *N* (%)
Enrolment	159/500 (31.8%)	114/332 (34.3%)	45/168 (26.8%)
Before delivery	208/500 (41.6%)	144/332 (43.4%)	64/168 (38.1%)
Month 3 post‐partum	244/500 (48.8%)	167/332 (50.3%)	77/168 (45.8%)
Month 6 post‐partum	265/500 (53.0%)	178/332 (53.6%)	87/168 (51.8%)
Month 9 post‐partum	280/500 (56.0%)	189/332 (56.9%)	91/168 (54.2%)
Month 12 post‐partum	294/500 (58.8%)	197/332 (59.3%)	97/168 (57.7%)
Total disclosed	294/500 (58.8%)	197/332 (59.3%)	97/168 (57.7%)

*Note*: Cumulative percentages of the visits at which women living with HIV had disclosed their HIV statuses to their male partners.

VS at 12 months post‐partum was not associated with HIV status disclosure at baseline (adjusted relative risk [aRR] 1.00; 95% CI: 0.80–1.26), baseline VS (aRR 1.17; 95% CI: 0.93–1.47) or male partner initiation of ART or PrEP (aRR 1.06; 95% CI: 0.84–1.34). We examined interactions between the woman's VL at baseline and male partner's initiation of PrEP/ART on maternal VS at 12 months post‐partum and found no effect modification (*p*‐value for interaction = 0.8). Female HIV status disclosure prior to partner enrolment was significantly associated with male partner HIV testing (HR 1.96; 95% CI: 1.49–2.58; *p* < 0.001) and ART/PrEP initiation (HR 1.70; 95% CI: 1.21–2.38; *p* = 0.002). Among women who did not disclose at baseline, male study participation predicted subsequent female disclosure (HR 2.10; 95% CI: 1.48–2.98; *p* < 0.001).

Of 472 women with pregnancy outcomes recorded, 434 (91.9%) gave birth to a live infant, 14 (3.0%) to a stillborn, 15 (3.2%) had a neonatal death and 9 (1.9%) had a miscarriage. Two infants (0.4%) were diagnosed with HIV at the 6‐month post‐partum visit (HIV incidence 1.46%; 95% CI: 0.18%–5.28% per year). For one infant, maternal VL was 887,000 copies/ml at this visit. For the other infant, maternal VL was 1040 and <50 copies/ml at the enrolment and 6‐month post‐partum visits, respectively. Most infants (425; 90.0%) were HIV negative, and 26 infants (5.5%) were not tested due to 15 neonatal deaths, 4 stillborn births and 7 live‐born infants lost to follow‐up.

Six women (1.2%) reported social harms, five in the HIVST arm and one in the SOC arm. Three cases were resolved after counselling and three resulted in partnership dissolution (Table S1).

## 4 DISCUSSION

In this RCT of secondary distribution of HIVST to male partners by PLWHIV in Uganda, which was compared to SOC invitation letters for fast‐track HIV testing at the study clinic, almost half (47%) of male partners were tested for HIV and two‐thirds of male partners started PrEP if HIV negative or ART if HIV positive. We observed no difference in partner HIV clinic testing rates by the proportion of male partners who initiated PrEP/ART, or in maternal VS at 12 months post‐partum. Two infants acquired HIV from mothers with detectable viremia. Encouragingly, the intervention was associated with very few social harms.

This study is unique in the focus on PWLHIV and linkage through partner testing to ART/PrEP. Prior studies of HIVST secondary distribution were conducted primarily among women without HIV who do not face potential social harms from disclosure and consequently have fewer barriers to approaching male partners. In addition, most prior studies relied on self‐report of HIVST distribution and partner testing, whereas our primary outcome was based on clinic‐based HIV testing, allowing ART/PrEP counselling and linkage‐to‐care. Almost half of the male partners tested for HIV; testing prevalence among men in Eastern Africa ranges from 9% in Madagascar to 81% in Rwanda [16]. Calling men in both arms if they did not come for testing a month after she enrolled, may have increased partner testing in the SOC arm and reduced the difference between arms. Our finding of similar partner testing rates in both arms differs from an RCT in Zambia (*n* = 116 PWLHIV) in which HIVST distribution by pregnant women increased male partner testing by 40% [17], and an RCT in Uganda where HIVST distribution doubled partner testing rates versus SOC [8]. However, these studies used women's self‐report of partner testing, whereas our study assessed male partner testing at the study clinic which is likely a more conservative measure of uptake of HIVST.

Antenatal clinics are key settings for reaching male partners and providing interventions that decrease HIV transmission risk. In East and Southern Africa, HIV testing and treatment gaps are larger among men than women for whom opt‐out HIV testing is part of routine clinical care [18]. A 2021 systematic review of 13 RCTs in SSA found that HIVST doubled testing uptake (RR 2.09; 95% CI: 1.69–2.58) relative to provider‐initiated testing [19]. Expanding access to HIVSTs, integrating HIV testing services (HTS) into clinics accessed by men, extending clinic operating hours and availing HTS in spaces patronized by men increases testing opportunities for asymptomatic men who rarely interact with the healthcare system.

Our finding that 88% of male partners with HIV started ART nearly aligns with the UNAIDS target of 95% of men with HIV initiating ART by 2025. Reaching men with HTS and linking them to care is essential to meeting global HIV goals as men are more likely than women to be diagnosed with advanced HIV disease [20] and need support for linkage to care and ART initiation after diagnosis. We observed no intervention effect on the linkage of male partners to ART/PrEP. Recent WHO guidance recommends ART initiation outside health facilities to decrease the substantial losses to care between community HIV testing and ART initiation [21]. A systematic review found that initiating ART outside health facilities increased the proportion of people starting ART, retained in care at 6–12 months and virally suppressed [21]. Differentiated service delivery for men established on ART, including less frequent visits and multi‐month dispensing, lowers transportation costs, decreases time spent waiting at clinic and reduces time lost from work [21], thus facilitating male engagement in care.

More than half of the men in this study tested HIV negative and were thus in serodifferent partnerships with PWLHIV. Approximately 60% of HIV‐negative men started PrEP. Men partnered with virally suppressed women received counselling about undetectable = untransmittable (U = U) and could opt out of taking PrEP if monogamous. Both men who seroconverted had female partners with suppressed VL suggesting HIV acquisition outside the study partnership. Men may acquire HIV from other partners during pregnancy/post‐partum. Genital inflammation/ulceration (adjusted hazard ratio [aHR] = 8.52), reporting ≥1 outside sexual partner (aHR = 3.86) and alcohol consumption (aHR = 3.84) significantly increased HIV acquisition risk among Zambian HIV serodifferent couples [22]. Reminding men that U = U is not protective outside the primary partnership is important, as almost half of the men reported another partner, and offering PrEP during this season of risk could reduce HIV acquisition in this group of men.

We found no association between female HIV status disclosure and VS. Studies of disclosure and VS in SSA PMTCT programmes have produced mixed results, with some studies showing benefit [23] and others finding no association [24]. Research from South Africa suggests a complex relationship between HIV status disclosure and VS [25], which is supported by our findings. Among women who disclosed their HIV status prior to their male partner's enrolment, disclosure nearly doubled male partner HIV testing and subsequent ART/PrEP initiation but was not associated with male linkage to care or maternal VS. Qualitative research suggests that women can maintain ART adherence within a wide range of disclosure behaviours [26].

PWLHIV reported concerns that providing either HIVST or an invitation letter could lead her partner to question her HIV status or result in loss of economic support, housing and social support if she disclosed. By study exit, the proportion of women who disclosed her status had almost doubled to 59%, which was similar by arm, and indicates the benefits of providing women with status disclosure support. Few social harms were reported by PWLHIV, perhaps because we screened out women with IPV history.

The strengths of our trial include the randomized design, large sample size, high retention and objective ascertainment of male partner outcomes (HIVST distribution, confirmatory testing and ART/PrEP initiation). Our study has limitations. PWLHIV were excluded if they reported IPV in the prior year so these results may not be generalizable to PMTCT programmes which do not screen women for IPV. As the HIVST arm allowed men to test at home, the proportion who tested may be underestimated by the number who came to the clinic for confirmatory HIV testing and study enrolment. Phone outreach may have biased the outcome of male partner testing to the null by increasing testing in the SOC arm, given that typically clinics do not actively conduct outreach to partners of PWLHIV.

## 5 CONCLUSIONS

In conclusion, our RCT of secondary distribution of HIVSTs by Ugandan PWLHIV to their partners found no intervention effect on male partner testing, male linkage to ART/PrEP or maternal VS at 12 months post‐partum. However, the findings that half of the male partners tested for HIV, two‐thirds of men started ART or PrEP and social harms were rare are encouraging. Additional strategies are needed to reach 95% of male partners testing for HIV and 95% linkage to care or prevention. Future studies should evaluate differentiated approaches to HIV testing and community ART initiation for men to achieve global targets for HIV epidemic control.

## COMPETING INTERESTS

AM reports a grant from Gilead Sciences, Inc. outside the submitted work, and was an advisor to ViiV Healthcare.

## AUTHORS’ CONTRIBUTIONS

AM, MB, GS, MS, DD and CLC conceptualized the manuscript. GS and DD conducted the statistical analyses. MB and AM wrote the first draft along with CLC. AM, MB, GS, MS, DD and CLC reviewed drafts and provided substantial edits. All authors read and approved the final version.

## FUNDING

This work was supported through a research grant from the United States National Institute of Mental Health (grant R01MH113434 to CLC). Study data were collected using REDCap, funded through grants UL1 TR002319, KL2 TR002317 and TL1 TR002318 from the National Center for Advancing Translational Sciences.

## DISCLAIMER

This paper represents the opinions of the authors and does not necessarily represent the official views of the National Institutes of Health.

## Supporting information


**Table S1**. Social Harm Narratives.

## Data Availability

The data that support the findings of this study are available from the corresponding author upon reasonable request.
