# Supplementary material for: Partner testing with HIV self‐test distribution by Ugandan pregnant women living with HIV: a randomized trial
Source: J Int AIDS Soc. 2023 Sep 7;26(9):e26156. doi: 10.1002/jia2.26156 (PMC10483500; doi:10.1002/jia2.26156)
Supplement: Supplementary file 1 — Table S1. Social Harm Narratives. [file JIA2-26-e26156-s001.docx]

**Supplementary Table 1. Social Harm Narratives**

| Arm | **Visit** | **Description** | **Result** | **Narrative updates** |
| --- | --- | --- | --- | --- |
| HIVST | First trimester | She took the HIVST kit to her partner and delivered it as expected. When she gave it to him, he used it, and it showed two lines [positive test]. He blamed her for infecting him with HIV. She got scared since he was very angry and left her matrimonial home. She lives with her sister who is very supportive. | Loss of income and economic support resulting in change of residence | Partnership dissolution |
| HIVST | Third trimester | The male partner was told his partner’s status, but he doubted it. He used the HIVST kit she gave him and obtained a negative result. He went to a clinic for confirmatory testing and again tested negative. He subsequently relocated from his house and disappeared. He did not buy the promised supplies for delivery and switched off his phone. She started a small business which sustains her financially. | Loss of income and economic support. | Partnership dissolution |
| SOC | Three months postpartum | She received an invitation letter to give her partner. When they came to the health center as a couple, they were received by a non-study staff member who disclosed her status to her partner without her consent. He subsequently changed his behavior towards her. Research staff provided couple counseling after which she reported a tremendous change in her partner's behavior. He ensured food was always available and he cared even more about the infant. He started planning for the baby's education. The couple’s relationship was restored after counseling. | Temporary hostility to female partner resolved after couple counselling | Couple reconciled |
| HIVST | Third trimester | Male partner verbally abused her after she gave him the kit and asked him to go with her to the research clinic. He became very angry and alleged that she no longer trusted him. He left home but later returned. She subsequently reported mutual understanding. | Temporary separation followed by resolution. | Couple reconciled |
| HIVST | Third trimester | Relationship break-up with loss of income and loss of economic support which resulted in change of residence. | He used the kit she gave him, and she said in jest that he was HIV positive. He reacted angrily. He returned after delivery and offered to support her financially. She also got another sexual partner who supports her. | Couple reconciled |
| HIVST | First trimester | Loss of income/economic support, change of residence | Her partner self-tested and found obtained a positive test. He ran away from home with the kit and never returned. His phone was switched off stopped financially supporting her. She started a small business selling merchandise to provide for herself. | Partnership dissolution |
